# Patterns of c-reactive protein RATIO response in severe community-acquired pneumonia: a cohort study

**DOI:** 10.1186/cc11291

**Published:** 2012-03-26

**Authors:** Luís M Coelho, Jorge IF Salluh, Márcio Soares, Fernando A Bozza, Juan Carlos R Verdeal, Hugo C Castro-Faria-Neto, José Roberto Lapa e Silva, Patrícia T Bozza, Pedro Póvoa

**Affiliations:** 1 Polyvalent Intensive Care Unit, Hospital de São Francisco Xavier, Centro Hospitalar de Lisboa Ocidental, Lisbon, Portugal (Hospital de São Francisco Xavier, CHLO, Estrada do Forte do Alto do Duque, 1449-005 Lisbon, Portugal; 2 CEDOC, Faculty of Medical Sciences, New University of Lisbon, Lisbon, Portugal (Campo dos Mártires da Pátria, 130 1169-056 Lisboa; 3 D'Or Institute for Research and Education, Rio de Janeiro, Brazil (Rua Diniz Cordeiro, n° 30 - Botafogo/RJ 22281-100 - Brasil; 4 Postgraduate Program, Instituto Nacional de Câncer, Rio de Janeiro, Brazil (Rua Riachuelo, 172 - Centro, Rio de Janeiro - RJ, 20230-014, Brasil; 5 Instituto de Pesquisa Clínica Evandro Chagas, FIOCRUZ, Rio de Janeiro, Brazil (Avenida Brasil, 4365, Manguinhos - Rio de Janeiro - RJ, 21040-360, Brasil; 6 Intensive Care Unit, Hospital Barra D'Or, Rio de Janeiro, Brazil (Avenida Ayrton Senna, 2541 - Barra da Tijuca, Rio de Janeiro - RJ, 22775-002, Brasil; 7 Laboratory of Immunopharmacology, Instituto Oswaldo Cruz, FIOCRUZ, Rio de Janeiro, Brazil (Avenida Brasil, 4365, Manguinhos - Rio de Janeiro - RJ, 21040-360, Brasil; 8 Pulmonary Diseases Department, Universidade Federal do Rio de Janeiro, Rio de Janeiro, Brazil (Ilha Cidade Universitária - Cidade Universitária, Rio de Janeiro - RJ, 21941-970, Brasil

## Abstract

**Introduction:**

Community-acquired pneumonia (CAP) requiring intensive care unit (ICU) admission remains a severe medical condition, presenting ICU mortality rates reaching 30%. The aim of this study was to assess the value of different patterns of C-reactive protein (CRP)-ratio response to antibiotic therapy in patients with severe CAP requiring ICU admission as an early maker of outcome.

**Methods:**

In total, 191 patients with severe CAP were prospectively included and CRP was sampled every other day from D1 to D7 of antibiotic prescription. CRP-ratio was calculated in relation to D1 CRP concentration. Patients were classified according to an individual pattern of CRP-ratio response with the following criteria: fast response - when D5 CRP was less than or equal to 0.4 of D1 CRP concentration; slow response - when D5 CRP was > 0.4 and D7 less than or equal to 0.8 of D1 CRP concentration; nonresponse - when D7 CRP was > 0.8 of D1 CRP concentration. Comparison between ICU survivors and non-survivors was performed.

**Results:**

CRP-ratio from D1 to D7 decreased faster in survivors than in non-survivors (p = 0.01). The ability of CRP-ratio by D5 to predict ICU outcome assessed by the area under the ROC curve was 0.73 (95% Confidence Interval, 0.64 - 0.82). By D5, a CRP concentration above 0.5 of the initial level was a marker of poor outcome (sensitivity 0.81, specificity 0.58, positive likelihood ratio 1.93, negative likelihood ratio 0.33). The time-dependent analysis of CRP-ratio of the three patterns (fast response *n *= 66; slow response *n *= 81; nonresponse *n *= 44) was significantly different between groups (p < 0.001). The ICU mortality rate was considerably different according to the patterns of CRP-ratio response: fast response 4.8%, slow response 17.3% and nonresponse 36.4% (p < 0.001).

**Conclusions:**

In severe CAP, sequential evaluation of CRP-ratio was useful in the early identification of patients with poor outcome. The evaluation of CRP-ratio pattern of response to antibiotics during the first week of therapy was useful in the recognition of the individual clinical evolution.

## Introduction

Community-acquired pneumonia (CAP) is a major cause of death due to infectious diseases in the Western world [1]. Among hospitalized patients, up to 36% require admission to the intensive care unit (ICU), and despite advances in antimicrobial therapy, more than one third of the patients will die during hospital stay [2-6].

After prescription of antibiotics, the evaluation of the individual clinical response and the assessment of resolution of pneumonia usually rely on the monitoring of the same criteria used for clinical diagnosis. Commonly used variables, such as temperature and white blood cell count, have a limited value in the assessment of clinical response to antibiotics [7,8] since both present significant improvements late in the course and can be influenced by drugs frequently used in ICU, such as steroids, antipyretics, or beta blockers.

To overcome these limitations, physicians frequently use serum biomarkers to assist in the clinical decision-making process, namely in the assessment of clinical response to antibiotic therapy. C-reactive protein (CRP) is one of these biomarkers and probably the most widely used [9]. In different infections and clinical settings, CRP discriminates, early in the clinical course, survivors from non-survivors. In addition, the course of relative CRP variations, the CRP ratio, after prescription of antibiotic therapy, can be classified in different patterns as fast response, slow response, and non-response [7,8]. Previous studies have demonstrated that the identification of the individual pattern of CRP ratio response to antibiotic therapy appears to be a reflection of the clinical course of infection independently of other possible confounders [7,8,10].

The aims of the present study were to evaluate the course of CRP ratio and identify the patterns CRP ratio response to antibiotic therapy during the first week in patients with severe CAP in order to differentiate between patients with good and poor outcome early in the clinical course and potentially provide a useful and easy-to-use tool for antibiotic stewardship.

## Materials and methods

### Study subjects

We conducted a prospective observational cohort study in two medical-surgical ICUs at tertiary hospitals (Barra D'Or Hospital, Rio de Janeiro, Brazil, and Garcia de Orta Hospital, Almada, Portugal). Patients with severe CAP that required ICU admission were consecutively included between November 2001 and December 2002 at Garcia de Orta Hospital and between August 2003 and June 2007 at Barra D'Or Hospital. To perform the present analysis, the independent databases were merged. The institutional review boards approved the study design and waived the need for informed consent. The present study was strictly observational and did not interfere in the decision-making process or clinical management. Patients were treated according to best standard ICU practice without any reference to the response patterns of CRP in their daily evaluation.

### Study design and definitions

Demographic, clinical, laboratory, and outcome data were prospectively collected. The Acute Physiology and Chronic Health Evaluation II (APACHE II) score was calculated after 24 hours of ICU admission [11]. Severe CAP was diagnosed according to the American Thoracic Society (ATS) criteria, and the CURB-65 scale - confusion of new onset (defined as an abbreviated mental test score of 8 or less), urea of greater than 7 mmol/L (19 mg/dL), respiratory rate of 30 breaths per minute or greater, systolic blood pressure of less than 90 mm Hg or diastolic blood pressure of 60 mm Hg or less, and age of at least 65 years - was used to evaluate its severity [12]. Patients were followed until death or hospital discharge. Antimicrobial therapy was prescribed in accordance with the ATS guidelines to all patients [12]. Patients with severe immunosuppression (for example, from solid organ or bone marrow transplant, HIV infection, or immunosuppressive treatment) and tuberculosis were excluded from the present study.

CRP, arterial oxygen tension/inspiratory oxygen fraction ratio (PaO_2_/FiO_2 _ratio), and Sequential Organ Failure Assessment (SOFA) [13] score were routinely measured during the first week of ICU stay at day 1 (D1), D3, D5, and D7. The CRP ratio was calculated in relation to the D1 CRP concentration. Patients were retrospectively classified with a modified version of previously defined CRP ratio patterns of the response to antibiotics [7,14]: fast response - when D5 CRP was not more than 0.4 of D1 CRP concentration; slow response - when D5 CRP was greater than 0.4 and D7 CRP was not more than 0.8 of D1 CRP concentration; or non-response - when D7 CRP was greater than 0.8 of D1 CRP concentration. Comparison between survivors and non-survivors was performed.

### Statistical analysis

Continuous variables are presented as the mean ± standard deviation unless stated otherwise. Comparisons between groups were performed by using the parametric unpaired and paired *t *test or the non-parametric Mann-Whitney *U *test and the Wilcoxon signed-rank test for continuous variables according to data distribution. The chi-squared test was used to carry out comparisons between categorical variables. Time-dependent analysis of different variables was performed via general linear model univariate repeated-measures analysis using a split-plot design approach. Receiver-operating characteristic curves were drawn for the D5 CRP ratio. The optimal CRP ratio cutoff was defined as the value associated with the highest sum of sensitivity and specificity (Youden's index).

To identify the variables (independent variables) predicting ICU outcome of severe CAP (dependent variable) during antibiotic therapy, a multivariable logistic regression model was constructed. Age, sex, APACHE II score, D1 PaO_2_/FiO_2 _ratio, mechanical ventilation, ICU-acquired infection, septic shock, D5 CRP ratio of greater than 0.5, and D1 SOFA score were included in the initial model to control for potential confounding factors. Backward stepwise variable elimination was then performed in order to develop the final model, and a *P *value of less than 0.05 was a requirement for acceptance. Model calibration and discrimination were assessed by using the Hosmer-Lemeshow goodness-of-fit test and the c statistic, respectively. Results are reported as adjusted odds ratio (AOR) with 95% confidence interval (CI). Data were analyzed by using PASW version 18.0 for MAC (SPSS, Inc., Chicago, IL, USA). All statistics were two-tailed, and the significance level was set at 0.05.

### Results

A total of 191 patients with severe CAP requiring ICU admission were included. The main characteristics of the study population are presented in Table 1. The ICU and hospital mortality rates were 21.9% and 24.6%, respectively. A microbiological diagnosis was established in 33 patients (17.2%) (Table 2). All patients with microbiological diagnosis had initial adequate antibiotic treatment. On ICU admission, 111 patients (58.1%) required invasive mechanical ventilation.

**Table 1:** Comparisons between survivors and non-survivors of severe community-acquired pneumonia

	All patients (*n *= 191)	Non-survivors (*n *= 42)	Survivors (*n *= 149)	*P *value^a^
Age in years	70 (54-81)	77 (64-83)	69 (53-79)	0.03
Male gender, number (percentage)	102 (53.4%)	19 (45.2%)	83 (55.7)	0.29
APACHE II score in points	15 (12-19)	18 (14-23)	15 (11-19)	0.004
CURB-65 in points	3 (3-4)	4 (3-5)	3 (2-4)	0.008
PaO_2_/FiO_2 _ratio in mm Hg	244 (172-295)	210 (157-266)	250 (177-300)	0.052
SOFA score in points	5 (3-8)	7 (3.7-10)	4 (3-7)	0.004
Positive blood cultures, number (percentage)	22 (11.5%)	7 (11.9%)	15 (11.4%)	0.99
Invasive mechanical ventilation, number (percentage)	111 (58.1%)	34 (80.9%)	77 (51.6%)	0.0007
Septic shock at ICU admission, number (percentage)	79 (41.3%)	26 (61.7%)	53 (35.5%)	0.0007
COPD, number (percentage)	41 (21.4%)	8 (19%)	33 (22.1%)	0.83
Diabetes, number (percentage)	36 (18.8%)	7 (16.6%)	29 (19.4%)	0.86
Asthma, number (percentage)	10 (5.2%)	2 (4.7%)	8 (5.3%)	0.81
Cardiac failure, number (percentage)	9 (4.7%)	1 (2.3%)	8 (5.3%)	0.69
C-reactive protein in mg/dL	15.3 (7.8-24.2)	14.3 (7.7-24.6)	15.5 (7.9-24.2)	0.91
ICU LOS in days	7 (4-15)	11.5 (5-22.7)	7 (4-13)	0.01
Hospital LOS in days	13 (9-26)	16 (6-36.7)	13 (9-23.5)	0.57

Results are expressed as median (25% to 75% interquartile range) unless stated otherwise. ^a^For comparisons among survivors and non-survivors. APACHE II, Acute Physiology and Chronic Health Evaluation II; COPD, chronic obstructive pulmonary disease; CURB-65 scale, confusion of new onset (defined as an abbreviated mental test score of 8 or less), urea of greater than 7 mmol/L (19 mg/dL), respiratory rate of 30 breaths per minute or greater, systolic blood pressure of less than 90 mm Hg or diastolic blood pressure of 60 mm Hg or less, and age of at least 65 years; ICU, intensive care unit; LOS, length of stay; PaO_2_/FiO_2 _ratio, arterial oxygen tension/inspiratory oxygen fraction ratio; SOFA, Sequential Organ Failure Assessment.

**Table 2:** Microorganisms isolated from the 33 (17.2%) patients with severe community-acquired pneumonia

Microorganism	*n *= 33
Gram-positive organisms	
*Streptococcus pneumoniae*	19
Methicillin-sensitive *Staphylococcus aureus*	
Group A *Streptococcus*	1
*Streptococcus agalactiae*	1
Gram-negative organisms	2
*Haemophilus influenzae*	3
*Pseudomonas aeruginosa*	3
*Klebsiella pneumoniae*	2
Atypical	
*Mycoplasma pneumoniae*	2

### D1 CRP and CRP ratio course from D1 to D7

At D1, CRP concentration was not significantly different between survivors and non-survivors (15.5 versus 14.3 mg/dL, *P *= 0.91). The course of CRP ratio during the first week of antibiotic therapy showed a steady and significant decrease in survivors (*P *= 0.01). As early as D3, the CRP ratio of survivors decreased by an average of 24% but by only 11% in non-survivors (*P *= 0.106). By D5 of antibiotic therapy, this divergent evolution of CRP ratio was markedly different; the CRP ratio was 0.46 (*P *< 0.001) in survivors but remained elevated in non-survivors, at 0.79 (*P *= 0.313). The ability of the CRP ratio by D5 to predict outcome assessed by the area under the receiver operating characteristic curve was 0.73 (95% CI = 0.64 to 0.82). By D5, a CRP concentration of above 0.5 of the initial level was a marker of poor outcome (sensitivity of 0.81, specificity of 0.58, positive likelihood ratio of 1.93, and negative likelihood ratio of 0.33).

In the multivariable analysis, only three variables - D1 SOFA score (per 1-point increment; AOR = 1.20, 95% CI = 1.06 to 1.37; *P *= 0.006), D5 CRP ratio of greater than 0.5 (AOR = 4.47, 95% CI = 1.64 to 12.20; *P *= 0.003), and mechanical ventilation (AOR = 9.39, 95% CI = 2.63 to 33.60; *P *= 0.001) - were independently associated with ICU mortality (model *n *= 175 and goodness of fit = 0.447).

### CRP ratio patterns of response to antibiotic therapy

**Figure 1:**
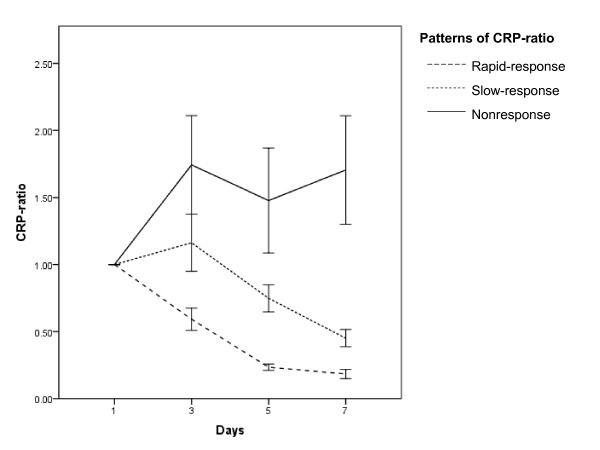
**Time-dependent analysis of C-reactive protein (CRP) ratio in different patterns of response during first week of antibiotic therapy**. Data are presented as the mean ± standard deviation.

Patients with severe CAP were retrospectively divided according to three patterns of the CRP ratio course during antibiotic therapy. Sixty-six (35%) patients were classified as fast response pattern, 81 (42%) as slow response, and 44 (23%) as non-response. The time-dependent analysis of CRP ratio (Figure 1) variations of the three different patterns was statistically different (*P *< 0.001). By D5, the CRP ratios were 0.23, 0.74, and 1.47 in patients exhibiting a fast response, a slow response, and a non-response pattern, respectively (*P *< 0.001).

When we analyzed the correlation between the CRP ratio patterns and outcome, we found marked differences in ICU mortality rate. Patients with the fast response, slow response, and non-response patterns presented ICU mortality rates of 4.6%, 17.3%, and 36.4%, respectively (*P *< 0.001) (Figure 1). Similarly, the hospital mortality was significantly different according to the CRP ratio pattern: fast response, 9.5%; slow response, 25.9%; and non-response, 43.2% (*P *= 0.001).

### CRP ratio patterns of response and clinical course

**Figure 2:**
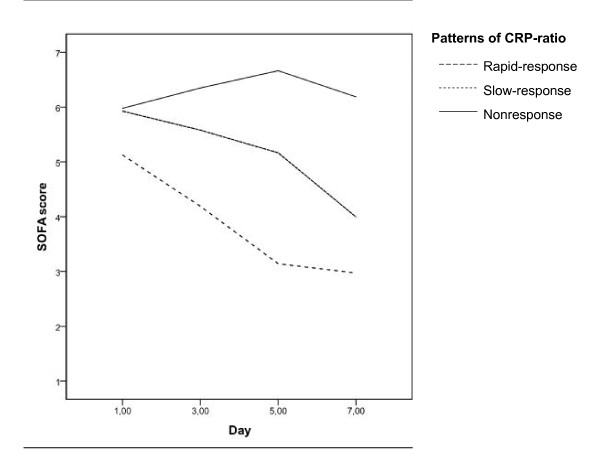
**Time-dependent analysis of the Sequential Organ Failure Assessment (SOFA) score during first week of antibiotic therapy in the different patterns of C-reactive protein (CRP) ratio**.

The clinical course during antibiotic therapy was monitored with SOFA score and the PaO_2_/FiO_2 _ratio. The time-dependent analysis of the PaO_2_/FiO_2 _ratio from D1 to D7 of antibiotic therapy in survivors and non-survivors was significantly different (*P *< 0.001) but was not different between the different patterns of CRP ratio (*P *= 0.210). However, the SOFA score from D1 to D7 of antibiotic therapy in survivors and non-survivors was significantly different (*P *< 0.001) as well as between the different patterns of CRP ratio response (*P *< 0.001) (Figure 2).

### Mechanically ventilated patients with CAP

In the subpopulation of mechanically ventilated patients (*n *= 111, ICU mortality of 26.1%), the time-dependent analysis of CRP ratio was also significantly different between survivors and non-survivors as well as between the different patterns of CRP ratio response to antibiotics (*P *= 0.002 and *P *< 0.001, respectively). In addition, in this subgroup of patients, the course of PaO_2_/FiO_2 _ratio during the first week of antibiotic therapy in the different patterns of CRP ratio response was not significantly different (*P *= 0.437). However, again, the SOFA score according to the different patterns of CRP ratio response during the same time period was significantly different (*P *= 0.001).

## Discussion

In the present study, we described the patterns of serial measurements of CRP ratio and its relation with clinical resolution of severe CAP in a large cohort of patients requiring intensive care admission. With the resolution of infection, the concentration of CRP decreases at a rate that is dependent on its half-life, since this marker exhibits a first-order elimination kinetics [15]. So the assessment of relative variations is more informative about the course of infection than absolute variations are [9,16]. In our study, survivors showed a continuous and significant decrease of CRP ratio during the first week of antibiotic therapy. Conversely, in non-survivors, CRP ratio remained elevated, and at D5, a CRP ratio of higher than 0.5 was associated with a fivefold increase in the risk of death in the ICU. Interestingly, in our study, patients with the non-response CRP ratio pattern presented a significantly higher ICU mortality than patients with fast or slow response patterns. Additionally, the identification of CRP ratio pattern of response to antibiotics, during the first week of therapy, was useful in the recognition of the individual clinical evolution of patients with severe CAP.

These data suggest that persistently elevated CRP values are indicative of poor response to antibiotic therapy. This could be the result of inadequate initial antibiotic therapy [7,8], the presence of other infectious complications [17], or a newly developed infection in another location [9,16]. Several studies have confirmed that serial measurements of CRP are useful in the monitoring of clinical course as well as assessment of patient outcome in different severe infections. In all of these studies, survivors by D3 to D4 presented values of CRP ratio of between 0.5 and 0.8 of the initial value [7,8,18-23]. This means that, after 72 to 96 hours of antibiotic therapy, the CRP of survivors decreases by 30% to 50% of the initial concentration. Besides, serial measurement of CRP allows the identification of various patterns of response to antibiotic therapy, as previously described by our group [7].

This concept of patterns of CRP ratio response to antibiotics has been tested in different clinical settings and reproduced by different research groups. In a recent study, Moreno and colleagues [20] studied the value of daily measurements of CRP in a cohort of 64 patients with nosocomial pneumonia. Patients were classified according to the CRP ratio in two groups: 'good' response (CRP ratios of lower than 0.67 at D10) and 'poor' response (non-response or biphasic response). The poor-response group (*n *= 34) had a mortality rate of 53% in comparison with 20% in the good-response group (*n *= 30) (relative risk = 2.65, 95% CI = 1.21 to 5.79; *P *= 0.01). Significant differences between the two groups were found on CRP ratios at D4 (*P *= 0.01). Also, in a cohort of 891 patients who had community-acquired sepsis and who were admitted to the ICU, Povoa and colleagues [19] found that patterns of CRP ratio response to antibiotics presented a marked correlation with hospital mortality. Patients with a non-response pattern had a 2.5 times higher probability of dying in comparison with patients with fast response (AOR = 2.5, 95% CI = 1.6 to 4.0; *P *< 0.001). Slow responders showed a non-significant increase on the odds of mortality in comparison with the fast responders (AOR = 1.5, 95% CI = 0.9 to 2.5; *P *= 0.124). The results of the present study are in agreement with this concept in the population of patients with severe CAP.

In all of these studies, the patterns of CRP ratio response allowed the early identification (between D3 and D4) of patients with poor response to antibiotics and consequently with poor prognosis. In our study, by D5, a CRP concentration of above 0.5 of the initial level was a marker of poor outcome.

The evaluation of changes in organ dysfunction/failure, assessed by the PaO_2_/FiO_2 _ratio and the SOFA score, could be helpful in the assessment of the effect of different therapeutic interventions. However, the PaO_2_/FiO_2 _ratio was not helpful in distinguishing between the different patterns of CRP ratio response during the first week of antibiotic therapy. The same was true in the subgroup of mechanically ventilated patients. Similar results were found in other studies in patients with ventilator-associated pneumonia and bloodstream infection [7,8].

When we studied the SOFA score from D1 to D7, we found a significant decrease in survivors in comparison with non-survivors. Also, patients with CRP ratio patterns of fast or slow response presented a significant decrease of SOFA score, whereas in patients with non-response, SOFA score remained almost unchanged. Similar findings were observed in the subgroup of mechanically ventilated patients. In our study, we show a good correlation between CRP ratio course and organ failure evolution measured by the SOFA score, either improving or not as well as the rate of improvement, respectively. In fact, the decrease of CRP ratio by D3 anticipates the decrease in SOFA score, and this could suggest that CRP is a better marker of resolution of severe CAP. Similar results were observed in patients with ventilator-associated pneumonia and bloodstream infections [7,8,24]. Additionally, the clinical application of the SOFA score is not as easy and straightforward as CRP interpretation, since SOFA score calculation implies the collection of data of several clinical and laboratorial parameters. So SOFA score determination is more time-consuming and difficult to perform routinely at the bedside. Consequently, the identification of the CRP ratio patterns of response in combination with the clinical evaluation could become a useful tool with good potential to reduce the length of antibiotic therapy as well as to reduce the risks of emergence of resistant strains and costs of medication, a hypothesis to be tested in future trials of CRP-guided therapy.

Our study has important strengths. It contained a reasonably large sample collected in two ICUs and evaluated the CRP ratio patterns of response to antibiotics in patients with severe CAP. However, there are limitations to the study; namely, it was an observational study. Also, the data came from two independent databases and were collected in different periods. As a result, we cannot exclude the possibility of time-dependent effect on the results. Most of the diagnoses were made on the basis of clinical and radiological criteria according to the ATS criteria, although only 17.2% of the patients were found to have microbiologically documented pneumonia. In addition, we have no information concerning antibiotic therapy previous to ICU admission. Besides, the attending physicians were not blinded to the CRP results. Finally, in this study, we evaluated only one biomarker, namely CRP. So we cannot exclude the possibility that similar results could be obtained with other biomarkers.

## Conclusions

Serial evaluation of CRP ratio was useful in the early identification of patients with severe CAP with a poor outcome. Besides, the recognition of the patterns of CRP ratio in patients with severe CAP provided additional information about the individual clinical course and this information could significantly influence the clinical decision-making process at the bedside. In patients with persistently elevated or rising CRP ratio (that is, a non-response pattern), an aggressive diagnostic and therapeutic approach should be attempted in order to prevent further clinical deterioration in an effort to change the poor associated prognosis. In contrast, patients with consistent CRP ratio decrease (that is, patterns of fast or slow response) usually have an adequate antibiotic therapy, rapid resolution of infection, and good prognosis.

## Key messages

• Serial evaluation of C-reactive protein (CRP) ratio is useful in the early identification of patients with severe community-acquired pneumonia (CAP) with a poor outcome.

• Recognition of the patterns of CRP ratio in patients with severe CAP provides information about the individual clinical course.

• In patients with a non-response pattern, an aggressive diagnostic and therapeutic approach should be attempted in order to change the poor associated prognosis.

## Abbreviations

AOR: adjusted odds ratio; APACHE II: Acute Physiology and Chronic Health Evaluation II; ATS: American Thoracic Society; CAP: community-acquired pneumonia; CI: confidence interval; CRP: C-reactive protein; D: day; ICU: intensive care unit; PaO_2_/FiO_2_: oxygen tension/inspiratory oxygen fraction; SOFA: Sequential Organ Failure Assessment.

## Competing interests

The authors declare that they have no competing interests.

## Authors' contributions

LMC, JIFS, and PP conceived the study, analyzed the data, and drafted the manuscript. All authors participated in the original design and in writing the original protocol and collected the data. All authors read and approved the final manuscript.
